# From Data to Insights: How Is AI Revolutionizing Small-Bowel Endoscopy?

**DOI:** 10.3390/diagnostics14030291

**Published:** 2024-01-29

**Authors:** Joana Mota, Maria João Almeida, Francisco Mendes, Miguel Martins, Tiago Ribeiro, João Afonso, Pedro Cardoso, Helder Cardoso, Patrícia Andrade, João Ferreira, Miguel Mascarenhas, Guilherme Macedo

**Affiliations:** 1 Precision Medicine Unit, Department of Gastroenterology, São João University Hospital, Alameda Professor Hernâni Monteiro, 4200-427 Porto, Portugalguilhermemacedo59@gmail.com (G.M.); 2 WGO Gastroenterology and Hepatology Training Center, Alameda Professor Hernâni Monteiro, 4200-427 Porto, Portugal; 3 Faculty of Medicine, University of Porto, Alameda Professor Hernâni Monteiro, 4200-427 Porto, Portugal; 4 Department of Mechanical Engineering, Faculty of Engineering, University of Porto, R. Dr. Roberto Frias, 4200-465 Porto, Portugal; j.ferreira@fe.up.pt; 5 Digestive Artificial Intelligence Development, R. Alfredo Allen 455-461, 4200-135 Porto, Portugal; 6 ManopH Gastroenterology Clinic, R. de Sá da Bandeira 752, 4000-432 Porto, Portugal

**Keywords:** artificial intelligence, convolutional neural network, deep learning, small bowel, capsule endoscopy, device-assisted enteroscopy

## Abstract

The role of capsule endoscopy and enteroscopy in managing various small-bowel pathologies is well-established. However, their broader application has been hampered mainly by their lengthy reading times. As a result, there is a growing interest in employing artificial intelligence (AI) in these diagnostic and therapeutic procedures, driven by the prospect of overcoming some major limitations and enhancing healthcare efficiency, while maintaining high accuracy levels. In the past two decades, the applicability of AI to gastroenterology has been increasing, mainly because of the strong imaging component. Nowadays, there are a multitude of studies using AI, specifically using convolutional neural networks, that prove the potential applications of AI to these endoscopic techniques, achieving remarkable results. These findings suggest that there is ample opportunity for AI to expand its presence in the management of gastroenterology diseases and, in the future, catalyze a game-changing transformation in clinical activities. This review provides an overview of the current state-of-the-art of AI in the scope of small-bowel study, with a particular focus on capsule endoscopy and enteroscopy.

## 1. Introduction

Artificial intelligence (AI) has been increasingly influential in our everyday life, and healthcare is not an exception, playing a significant role in specialties with strong imaging and diagnostic components [1]. Undoubtedly, the awareness of AI’s innumerable opportunities in the medical field is growing exponentially, mainly because of its potential to positively transform healthcare, leading to significant advancements. Combined with the increasing pressure from patients and countries to improve healthcare quality without escalating the costs, AI seems to play a crucial role in facilitating this goal [2].

AI is a broad descriptor that refers to the development and application of computer science that is capable of performing tasks that usually require human intelligence [1]. At present, society is witnessing the dominance of big data, characterized by the five Vs: volume, value, velocity, variety, and veracity. In gastroenterology, the massive collection of digital photos and medical information provides an unmatched combination of resources for machine learning (ML) and deep learning (DL) technologies [2,3].

ML is a subset of AI and is defined as the ability of an algorithm to learn new tasks through data analysis without being specifically programmed to. It can be either supervised or unsupervised; the differences between both are beyond the spectrum of this article [2,4,5].

DL is a transformative subset of ML that resembles the functioning of the human brain and enables the handling of more complex tasks. It uses a backpropagation algorithm consisting of multiple layers, allowing the system to adjust each layer’s parameters based on the preceding layers’ representations and provide output more efficiently. The key advantage lies in the possibility of knowledge transfer acquired from a pre-trained model on one task to be applied to a new task, eliminating the need to design a new model for each individual task [2,4,5].

Convolutional neural networks (CNNs) are the most prominent DL technique, inspired by the organization of the human visual cortex and, therefore, explicitly tailored towards image and pattern recognition. By simulating the connectivity pattern between neurons responding to overlapping regions in the visual field, CNNs require less preprocessing and rely less on prior knowledge or human effort [2,4,5]. 

The small bowel (SB) has long been considered by endoscopists as a technical challenge due to its length and complex anatomy [6]. In the last two decades, the study of the SB was revolutionized by the development of capsule endoscopy (CE) and device-assisted enteroscopy (DAE), representing a decisive breakthrough in managing SB diseases [7]. 

This review provides an overview of the current reported applications of AI in the scope of SB study, with a particular focus on capsule endoscopy (CE) and device-assisted enteroscopy (DAE). 

## 2. Application in Small-Bowel Capsule Endoscopy

**Figure 1:**
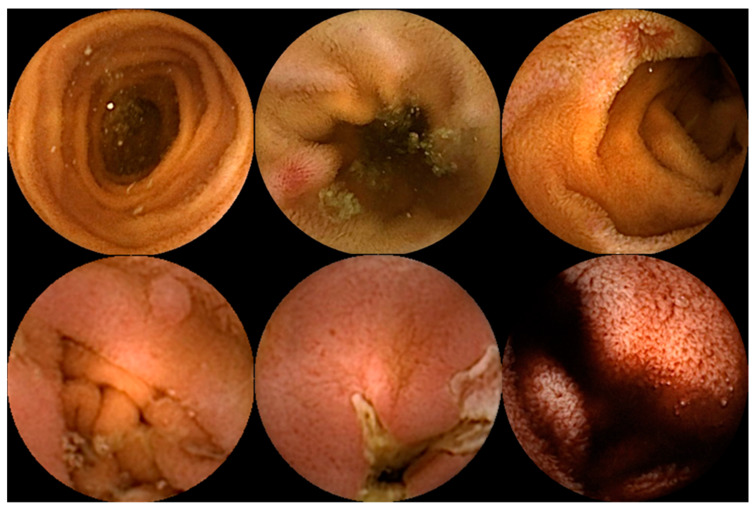
Capsule endoscopy images of several small-bowel pathologies. In the top row, the left image corresponds to normal mucosa, and the two images on the right illustrate vascular lesions. In the bottom row, the left image corresponds to a protruding lesion, the center image to an ulcer, and the right image to hematic residues.

Capsule endoscopy allows for a non-invasive and painless evaluation of SB mucosa, essentially being a diagnostic modality [5,7]. This exam is fundamental to the diagnosis of obscure gastrointestinal bleeding (OGIB) but also the study of Crohn’s disease (CD), SB tumors, celiac disease (CeD) (extent and severity), and others [5,7,8], as illustrated in Figure 1. However, it is essential to note that CE has some drawbacks. Among these is the dependence on the examiner’s clinical experience and the time and labor involved in the image review process (previous series have reported reading times of over 40 to 50 min), which makes it a task prone to error [9,10]. Therefore, AI will probably contribute to minimizing these limitations and increase its potential. Nowadays, this topic is becoming more popular, resulting in an increasing number of recent research articles dedicated to it. Below, we summarize the primary evidence regarding this subject. 

### 2.1. AI and Obscure Gastrointestinal Bleeding

Gastrointestinal (GI) bleeding can originate anywhere from the mouth to the rectum or anus [11]. We classified GI bleeding into upper, lower, and middle based on the location; the markers are anatomical landmarks: the ampulla of Vater and terminal ileum. Even though they usually have different presentations, overlap can occur, making it challenging to identify the bleeding source [12]. OGIB refers to GI bleeding from an unidentified origin that persists despite a comprehensive upper and lower GI evaluation with an endoscopic evaluation of the terminal ileum. OGIB is divided into obscure overt or obscure occult based on the presence or absence of clinically visible bleeding, respectively [11,12].

Different etiologies can cause OGIB, with angioectasias or nonsteroidal anti-inflammatory drug-induced ulcers being the most common in older patients (>40 years). The approach to studying different etiologies depends on whether it is overt or occult, whether signs of severe bleeding are present, and whether the patient is fit for endoscopic evaluations [11,13]. In general, video capsule endoscopy (VCE) is the first diagnostic step in OGIB, in the absence of contraindications such as obstruction. Indeed, OGIB is the most common indication for CE [14]. When the bleeding site is identified during VCE, specific treatment should be initiated.

Many research articles have been dedicated to this field since at least 2007, mainly because of its prevalence in clinical activity. Table 1 summarizes the main results of multiple studies about AI models’ application in the study of the SB bleeding lesions. In 2009, Pan et al. developed a CNN to detect a bleeding image using color texture features. This research used a total of 150 full CE videos, 3172 bleeding images, and 11,458 non-bleeding images to test the algorithm, achieving a sensitivity and specificity at the image level of 93.1% and 85.6%, respectively. This study achieved better results than the previous research and used a much larger dataset [15].

Fu et al. wanted to overcome some of the limitations of the suspected blood indicator, which included the ability to only detect active bleeding and the method’s insufficient sensitivity and specificity. For that, they created a computer-aided design method based on a support vector machine that detects bleeding regions with high sensitivity, specificity, and accuracy (99%, 94%, and 95%, respectively). They also used a different image analysis method, grouping pixels based on color and location through superpixel segmentation, which reduced the computational complexity [16]. Later, Jia et al. developed a deep CNN to automatically detect bleeding in wireless capsule endoscopy (WCE) images. They compared their method with Fu et al. and others, achieving better results [17].

Fan et al. used the AlexNet CNN to detect ulcers and erosions in SB mucosa. This study reported an AUC ROC curve over 0.98 in ulcer and erosion detection and an accuracy of 95.16% and 95.34%, a sensitivity of 96.80% and 93.67%, and a specificity of 94.79% and 95.98%, respectively. This research was pioneered by using DL to assess two different lesions simultaneously [18]. In the following year, Aoki et al. also trained a deep CNN to detect erosions and ulcerations in WCE images automatically. The model reported an AUC of 0.958 and a sensitivity, specificity, and accuracy of 88.2%, 90.9%, and 90.8%, correspondingly [19].

Wang et al. applied a deep learning framework to ulcer detection using a large dataset (1504 patient cases—1076 with ulcers, 428 normal). The results of this study were moderately decent and indicate a strong correlation between ulcer size and detection [20].

Aoki et al. developed an unprecedented CNN method to assess if a CNN can reduce endoscopists’ reading time (trainees and experts). To achieve this, they compare the reading times and detection rates of mucosal breaks (erosions or ulcerations) between endoscopist-alone readings (process A) with endoscopist readings after a first screening by the CNN (process B). They used 20 full videos and reported a significantly shorter duration for process B (expert: 3.1 min; trainee: 5.2 min vs. expert: 12.2 min; trainee: 20.7 min) without compromising the detection rate of mucosal breaks. This study reinforces these methods’ importance and practical application in clinical settings [21]. The same author recently developed a CNN capable of detecting blood in the SB lumen using 27,847 images from 41 patients (6503 images depicting blood content from 29 patients and 21,344 images of normal mucosa from 12 patients). They compared the performance of the CNN with the suspected blood indicator (SBI), achieving significantly higher sensitivity, specificity, and accuracy, corresponding to 96.63%, 99.96%, and 99.89%, than the SBI. This study suggests that a CNN could outperform the SBI software already used in real-time practice [22].

Ghosh developed a deep transfer learning framework for automated bleeding detection and bleeding zone identification in CE images, achieving satisfactory global accuracy [23]. More recently, a Portuguese group created a CNN-based algorithm that automatically detects blood and hematic residues within the SB lumen in CE images. Throughout three stages of development, the model’s accuracy demonstrated a tendency to increase as data were repeatedly loaded into the multi-layer CNN. In the last stage, it achieved an area under the ROC curve of 1.0, a sensitivity of 98.3%, a specificity of 98.4%, and an accuracy of 98.2%, with excellent reading times (186 frames/second) [14]. For the first time. the same group more recently developed a CNN capable of automatically identifying and classifying multiple SB lesions with different bleeding potential, using a dataset of 53,555 images of a total of 5793 CE exams from 4319 patients of two different medical centers. Each frame was evaluated for the type of lesion, lymphangiectasia, xanthomas, vascular lesions, ulcers, erosions, protruding lesions, and luminal blood, and the hemorrhagic risk was evaluated based on Saurin’s classification (P0, P1, and P2, for lesions without hemorrhagic potential, or with intermediate or high hemorrhagic potential, respectively). This research reported sensitivity and specificity for the automatic detection of various abnormalities, approximately 88% and 99%, respectively. It also reported high sensitivity and specificity for detecting P0, P1, and P2 lesions. This study is particularly interesting because it sets a precedent for future advancements in this area, likely contributing to real-time implementation [24].

### 2.2. AI and Vascular Lesions 

Angioectasia is the most common vascular lesion in the GI tract and results from the formation of aberrant blood vessels. This lesion is the cause of more than 8% of bleeding episodes, and its prevalence is highly linked to advanced age [5,11,25]. Previous studies mainly focused on detecting bleeding lesions rather than specifically angioectasia lesions. In 2012, a study reported that only approximately 70% of angioectasias were detected by experts, highlighting the urgent need for improvement [25]. AI could be a tool with significant potential in this regard. The primary findings of several research studies on the use of AI models in the investigation of SB angioectasias are compiled in Table 2.

Indeed, in 2016, Vieira et al. developed an automatic segmented method capable of identifying angioectasias using different color spaces [26]. At a later stage, the same group improved the segmentation algorithm used in prior research, outperforming the last study and achieving sensitivity and specificity values of over 96% [25]. Later, Noya et al. developed a system of automatic angioectasia lesion detection using color-based, texture, statistical, and morphological features. This study reported a sensitivity of 89.51%, a specificity of 96.8%, and an AUC value of 82.33% [27]. Leenhardt et al. developed a CNN model that could automatically detect and localize angioectasias, using 6360 images from 4166 CEs and achieving a sensitivity and specificity of 100% and 96%, respectively [28].

Subsequently, further studies were conducted using CNNs. Tsuboi et al. developed a deep CNN system for the automatic detection of SB angioectasia in CE still images using 2237 still frames of CE, achieving an AUC of 0.998, and the sensitivity, specificity, positive predictive value, and negative predictive value of the CNN was 98.8%, 98.4%, 75.4%, and 99.9%, respectively [29]. More recently, Chu et al. developed a DL algorithm that used ResNet50 as a skeleton network to segment and recognize angioectasia lesions (angioectasia, Dieulafoy’s lesion, and arteriovenous malformation). This study used a dataset of 378 patients and comprised a test set with 3000 images, which contained 1500 images without lesions and 1500 images with lesions. They compare their model network with others available (PSPNet, Ground truth, DeeplabV3+, and UpperNet), achieving an accuracy of 99%, a mean intersection over union of 0.69, a negative predictive value of 98.74%, and a positive predictive value of 94.27% [30].

**Table 2:** Summary of studies on AI application in the study of vascular lesions. Ref, reference; Pub, publication year; S, sensitivity; Sp, specificity; PPV, positive predictive value; NPV, negative predictive value; Acc, accuracy; AUC, area under the curve; SB, small bowel; GI, gastrointestinal; CNN, convolutional neural network; ML, machine learning.

Author Ref.	Field	Pub. Year	Study Design	Aim	Number of Subjects	Training Dataset	Validation and Testing Dataset	AI Type	Results
Small-Bowel Capsule Endoscopy									
Vieira [25]	Angioectasia	2019	Retrospective	Detect SB angioectasia	-	Two different datasets		ML	S and SP over 96%
Noya et al. [27]	Angioectasia	2017	Retrospective	Detect SB angioectasia	799 lesion frames and 849 normal frames from 36 patients	514 regions with lesion and 22,832 regions with no lesion	514 regions with lesion and 22,832 regions with no lesion	CNN	S: 89.5%. Sp: 96.8%
Leenhardt et al. [28]	Angioectasia	2019	Retrospective	Detection of SB angioectasias	4166 videos	300 GI angioectasia images and 300 normal images	300 GI angioectasia images and 300 normal images	CNN	S: 100%. Sp: 96%
Tsuboi et al. [29]	Angioectasia	2020	Retrospective	Detection of SB angioectasias	189 patients	2237 GI angioectasia from 141 patients	488 images of angioectasia and 10,000 normal images from 48 patients	CNN	AUC 0.998 S: 98.8% Sp: 98.4%
Chu et al. [30]	Angioectasia	2023	Retrospective	Detect SB angioectasias (angioectasia, Dieulafoy’s lesion, and AV malformation)	378 patients	7393 lesion images	1500 lesion images 1500 normal images	CNN	Acc: 99%. NPV: 98.7% PPV: 94.3%

### 2.3. AI and Protruding Lesions

CE plays an essential role in investigating patients with clinically or imaging-suspected SB tumors, as well as in monitoring patients with hereditary polyposis syndromes [31]. SB protuberant lesions consist of various pleomorphism lesions, in which tumors of SB are evidently included. Their detection is challenging due to the lesions’ pleomorphism [32]. Table 3 provides an overview of the key data pertaining to the application of AI in protruding lesion detection.

Barbosa and co-workers, in 2008, developed an algorithm based on the textural analysis of the different color channels capable of detecting tumor lesions. They used a small dataset and reported 98.7% sensibility and 96.6% specificity in detecting tumor lesions in the SB [33]. The same group also developed an algorithm based on combined information from both the color and texture of the images for the detection of tumors of the SB. This algorithm was based on the previous study from the same authors, but this one used a more extensive dataset. It also achieved excellent performance, 93.1% specificity, and 93.9% sensitivity [34].

Li et al. also used an algorithm based on shape features, but it only used data retrieved from two patients, which limits its applicability to real practice [35]. The same authors also performed a comparative study using a computer-aided system for detecting tumors in CE images through a comparative analysis of four texture features and three color spaces. The best performance achieved was an average accuracy of 83.50% and a specificity and sensitivity of 84.67% and 82.33%, respectively. They concluded that different color spaces have different impacts on the computer-aided system’s performance [36]. In the following year, the same group developed a computerized tumor detection system for CE images. Using texture features and a support vector machine, they achieved an accuracy of 92.4% [37]. Other studies only detected tumors in the SB [38,39]. Yuan et al. developed a computer-aided detection method to recognize polyp images and other structures (bubbles, turbid images, and clear images) in CE images with an average accuracy of 98%. This study reinforces that luminal content makes it difficult to evaluate frames [40].

More recently, Saito and co-workers developed, for the first time, a CNN capable of identifying and classifying protruding lesions (polyps, nodules, epithelial tumors, submucosal tumors, and venous structures) in CE images using a large dataset. This research achieved an overall AUC of 0.911 and a sensitivity and specificity of 90.7% and 79.8%, respectively. This method brings the algorithm much closer to real clinical practice, enhancing its practicality in clinical settings [41].

Saraiva et al. developed a pioneer CNN designed to automatically detect SB protruding lesions and evaluate the lesions’ hemorrhagic potential. Using 1483 CE exams, a total of 18,625 images were extracted, with 2830 images showing protruding lesions and the rest showing normal mucosa. Each frame was evaluated for enteric protruding lesions (polyps, epithelial tumors, subepithelial lesions, and nodules), and the hemorrhagic potential was estimated according to Saurin’s classification. Overall, the model achieved an accuracy of 92.5%, a sensitivity and a specificity of 96.8% and 96.5%, respectively, and an excellent reading time (70 frames per second) [32].

**Table 3:** Summary of studies on AI application in the study of protruding lesions. Ref, reference; Pub, publication year; S, sensitivity; Sp, specificity; Acc, accuracy; SB, small bowel; CE, capsule endoscopy; WCE, wireless capsule endoscopy; SVM, support vector machine; CNN, convolutional neural network; SSAEM, stacked sparse autoencoder with image manifold constraint; MLP, machine learning perceptron.

Author Ref.	Field	Pub. Year	Study Design	Aim	Number of Subjects	Training Dataset	Validation and Testing Dataset	AI Type	Results
Small-Bowel Capsule Endoscopy									
Barbosa et al. [33]	Protruding lesions	2008	Retrospective	Detection of SB tumors	-	104 tumor images 100 normal images	92 tumor images 100 normal images	MLP	S: 98.7% Sp: 96.6%
Barbosa et al. [34]	Protruding lesions	2012	Retrospective	Detection of SB tumors	700 tumoral frames and 2300 normal frames	-	-	MLP	S: 93.1% Sp: 93.9%
Li et al. [35]	Protruding lesions	2009	Retrospective	Detection of SB tumors	150 abnormal images and 150 normal images from 2 patients	-	-	MLP	S: 89.8% Sp: 82.5% Acc: 86.1%
Li et al. [36]	Protruding lesions	2011	Retrospective	Detection of SB tumors	600 images of tumors and 600 normal images from 10 patients	540 normal images and 540 tumor images from 9 patients	60 normal images and 60 tumor images from 1 patient	SVM	S: 82.3% Sp: 84.7% Acc: 83.5%
Li et al. [37]	Protruding lesions	2012	Retrospective	Detection of SB tumors	600 images of tumors and 600 normal images from 10 patients	-	-	SVM	Acc: 92.4%
Yuan et al. [40]	Protruding lesions	2017	Retrospective	Polyp detection	1000 polyp images and 3000 normal images	-	-	SSAEM	Acc: 98%
Saito et al. [41]	Protruding lesions	2020	Retrospective	Identify and classify protruding lesions	-	30,584 WCE images of protruding lesions from 292 patients	7507 images of protruding lesions from 93 patients and 10,000 normal images	CNN	S: 90.7% Sp: 79.8%
Saraiva et al. [32]	Protruding lesions	2021	Retrospective	Detect SB protruding lesions and evaluate the lesions’ bleeding potential	1483 CE exams from 1229 patients. 18,625 images extracted	14,900 images (2264 images of protruding lesions and 12,636 images of normal mucosa)	3725 images of protruding lesions, and 3159 images with normal mucosa	CNN	S: 96.8% Sp: 96.5% Acc: 92.5% Reading time 70 frames per second

### 2.4. AI and Pleomorphic Lesion Detection

Most of the currently developed advanced systems can only detect one type of lesion at a time, which does not meet the requirements for clinical practice implementation [42]. Therefore, there has been a need to develop algorithms capable of detecting multiple pathologies in a single examination. Table 4 presents an overview of several studies exploring the use of AI in pleomorphic lesion detection. 

Ding and co-workers developed a CNN algorithm capable of classifying various lesions in SB CE images, unlike previous studies focusing only on specific lesions. This study used an extensive multicenter dataset—data from 6970 patients (158,235 images from 1970 cases used in the training phase and 5000 cases in the validation phase)—to screen out different lesions (abnormal lesions and normal variants). The algorithm reported excellent performance and time efficiency, with a mean reading time of approximately 6 min compared with conventional reading times of 97 min [43].

The latter study was followed by other research studies using CNNs to detect a variety of mucosal abnormalities. Otani et al. trained the deep neural network system RetinaNet to diagnose various SB lesions using a training dataset of 167 patients (398 images of erosions and ulcers, 538 images of angioectasias, 4590 images of tumors, and 34,437 normal images from 11 patients), achieving an AUC value for tumors, erosions and ulcers, and vascular lesions of 0.950, 0.996 and 0.950, respectively [44]. Aoki and co-workers conducted prior research on detecting individual abnormalities. In this article, they developed a deep CNN system capable of detecting various abnormalities and compared it with the QuickView mode, also reporting excellent results [45]. Vieira et al. applied multi-pathology classification and segmentation to the KID dataset. The model reported good performances in lesion detection and segmentation tasks, suggesting that these two should be used together in future works [46]. Furthermore, Hwang et al. developed a CNN capable of automatically detecting various SB lesions (hemorrhagic and ulcerative lesions). They trained the CNN in two ways: the combined model (separately identifying hemorrhagic and ulcerative lesions and then combining the results) and the binary model (identifying abnormal images without discrimination). Both models achieved high accuracy for lesion detection, and the difference between the two models was not significant. However, the combined model reported results with higher accuracy and sensitivity [42].

### 2.5. AI and Small-Bowel Compartmentalization

AI has tremendous potential in assisting with the localization of CE within the GI tract and could decrease the time required to identify organic boundaries, which is necessary for studies of automatic lesion detection and locating lesions in clinical practice [47]. 

Prior to 2017, many research articles aimed to locate the pylorus. However, they achieved neither excellent accuracy nor excellent reading times. In turn, Wang et al. developed an SVM method that was able to achieve that aim, using 3801 images from the pyloric region, 1822 from the pre-pyloric region, and 1979 from the post-pyloric region. The study reported an accuracy of 97.1% and a specificity of 95.4% in a time-efficient manner (1.26 min on average) [47].

### 2.6. AI and Celiac Disease

CeD is an immune-mediated disorder known for being a gluten-sensitive enteropathy. The diagnosis relies on a sequential approach and a combination of clinical features, serology, or histology. Biopsy was, for a long time, considered the ‘gold standard’ for diagnosing CeD and is still mandatory in most cases [48]. Despite no substitute for duodenal biopsies, CE seems to be a promising alternative for diagnosing CeD, excluding other diagnoses, and evaluating the extent of the disease. Table 5 is a summary on the main evidence regarding the use of AI for celiac disease, both for diagnosis and severity grading.

Ciaccio et al. developed a threshold classifier able to predict CeD based on images. Using image data from eleven CeD patients and ten control patients and analyzing nine different features, they reported a threshold classifier with 80% sensitivity and 96% specificity [8].

Later, Zhou et al. developed a CNN to evaluate the presence and degree of villous atrophy objectively. The training set had CE videos from six CeD patients and five controls, and each frame was rotated every 15 degrees to form a new candidate proposal for the training set, which improved the sensitivity and specificity. The authors achieved a 100% level of sensitivity and specificity in the testing set. This study introduced a new prospect, the automatic correlation between Marsh classification and video capsule images [49].

Koh et al. used a combination of various image features to classify normal or CeD images using a computer-aided detection (CAD) system. The study reported an accuracy level of 86.47%, and a sensitivity and specificity of 88.43% and 84.60%, respectively. This study reinforced that the CAD system can improve and change how we diagnose CeD [50].

In 2020, Wang et al. developed a CNN system that combined different techniques, utilizing data from 52 CeD videoclips and 55 healthy videoclips. Overall, it achieved remarkable results in the diagnosis of CeD, with accuracy, sensitivity, and specificity of 95.94%, 97.20%, and 95.63%, respectively. This study highlights the role of integrating different technologies to achieve better results and robustness [51].

More recently, Stoleru et al. presented an algorithm proving that computer-aided CeD detection is possible even without using complex algorithms. They processed images with two modified filters to analyze the intestinal wall’s texture, proving that a diagnosis can be obtained through image processing and without complex algorithms [52]. Also, Chetcuti Zammit et al. developed an ML algorithm capable of quantitatively grading CeD severity. They used a training dataset of 334,080 frames from 35 patients with biopsy-proven CeD and 110,579 frames from 13 patients without CeD. A strong correlation was observed between the celiac severity scores provided by the algorithm and the average expert reader scores. This study used a large patient cohort, suggesting reproducibility in real time [53].

### 2.7. AI and Inflammatory Bowel Activity

Inflammatory bowel disease (IBD) has ulcerative colitis and CD as its principal forms. Approximately 70–90% of CD patients have SB disease [54]. The role of capsule endoscopy in IBD disease or suspected disease, particularly in CD, is well-established for both diagnosis and follow-up in a non-invasive way [55]. Quantitative scores, namely the Lewis score and the capsule endoscopy CD activity index, are commonly used in medical practice to quantify mucosal inflammation during CE. However, these scores rely on intra-examiner variability. Therefore, AI can play a significant role in reducing the limitations of CE and minimizing the intra-observer variability and, by doing so, improve clinical practice and minimize the risk and cost [56,57]. Table 6 aims to compile the most relevant research on AI implementation for IBD diagnosis and grading. In Table 6, a summary of studies on AI application in the study of inflammatory bowel is described.

Klang et al. developed a CNN to classify images as normal mucosa or mucosa with ulcerations and aphthae. They used data from 49 patients, with 17,640 CE images in total. The model reported an excellent AUC, above 0.94, in detecting ulcerations in patients with CD [56]. In the same year, Barash et al., in collaboration with Klang, developed a DL algorithm capable of detecting and grading the severity of ulcers in CD. They divided the study into two parts. In the first part, 1108 pathological CE images were graded from 1–3 according to ulcer severity by two evaluators. Also, the inter-reader variability was calculated, reporting an overall inter-reader agreement of only 31% for the images (345/1108). In the second part, Barash and co-workers used a CNN to classify the ulcers’ severity automatically. They achieved an overall agreement between the consensus reading and the automatic algorithm of 67% (166 /248). This study was the first to use AI to assess ulcer severity rather than a binary classification (ulcer vs. normal) [58].

The presence of ulcers suggests a worse prognosis for the disease, but the presence of strictures also does. As a result, Klang and co-workers recently tested a DL network capable of detecting CE images of strictures in CD. They used a dataset of 27,892 CE images (1942 stricture images, 14,266 normal mucosa images, and 11,684 ulcer images). Overall, the algorithm reported an average accuracy of 93.5% in detecting strictures and excellent differentiation between strictures, normal mucosa, and different grades of ulcers [59].

### 2.8. AI and Small-Bowel Cleansing

**Figure 2:**
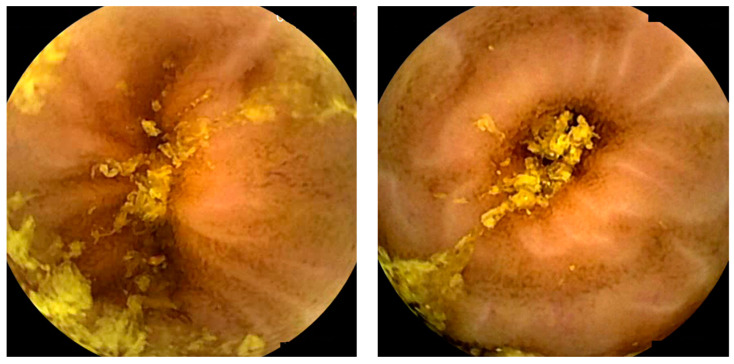
Images depicting the quality of small-bowel preparation. The left image corresponds to a satisfactory preparation. The right image corresponds to an excellent preparation.

Properly evaluating images from CE requires a well-prepared bowel, which requires the absence of air bubbles, bile, and intestinal debris. A high-quality preparation ensures optimal visualization of the mucosa and allows the drawing of meaningful and reliable conclusions. This is particularly important in CE because the endoscopist has no control over the field of view, as illustrated in Figure 2. Furthermore, there is currently no established gold standard for intestinal CE preparation due to the lack of objective and automated methods for evaluating cleansing [10,60,61]. 

Nowadays, there are both operator-dependent scores, such as Brotz and Park, and automated scores to evaluate intestinal CE preparation. The automated scores are considered objective, reliable, and reproducible, thereby overcoming the limitations of operator-dependent scores [62]. Table 7 compiles key studies on AI adoption for evaluating SB cleanliness quality. 

Van Weyenberg et al. developed a computed assessment able to assess the quality of SB preparation using the PillCam^®^ CE system (Medtronic, Dublin, Ireland), based on the color intensities in the red and green channel of the tissue color bar (visible mucosa is associated with red colors, whereas fecal contamination lumen is associated with green colors). Comparing this method with three previous quantitative and qualitative scores, they found a high overall agreement, indicating that this method should be integrated into video CE reading [63]. Later, Ponte et al. adapted this computed assessment to the MiroCam^®^ CE system (Intromedic, Seoul, South Korea); the results were found to be inferior to those reported by Van Weyenberg. However, it remained statistically significant, reinforcing the practicality of the automated score in different CE systems [64]. Abou Ali et al. also adapted this method, using the PillCam^®^ CE system, achieving a sensitivity of 91.3% and a specificity of 94.7%, reinforcing that this computed assessment score had the potential for automated cleansing evaluation [65]. Later, similar computed scores were created: Oumrani et al. used a multi-criteria computer-aided algorithm with three parameters tested individually or combined: the red/green ratio, an abundance of bubbles, and brightness. The main objective was to assess the quality of SB visualization in third-generation still frames, achieving a sensitivity and a specificity of 90% and 87.7%, correspondingly. These results were obtained with optimal reproducibility [66].

More recently, studies in this area have been conducted utilizing DL algorithms. Noorda et al. developed a CNN capable of automatically evaluating the cleanliness of the SB in CE, classifying images as dirty or clean, using a dataset of over 50,000 images. They compared their algorithm with other algorithms that have more parameters, and their algorithm achieved an excellent balance of performance/complexity. They also compared the results of two medical specialists achieving acceptable agreement, with κ1 values of 0.643 and 0.608, corresponding with specialists one and two [60]. Leenhardt et al. developed a CNN algorithm capable of assessing SB cleanliness during CE. This method reported a high sensitivity but a moderate specificity and a reading time of 3 ± 1 min [67].

Nam et al. developed a software for calculating cleansing scores for the SB using a DL method. The training dataset consisted of a five-step scoring system based on mucosa visibility (five being more than 90% of mucosa visible, and one being less than 25% of mucosa visible). This score was compared to a clinical assessment evaluation by gastroenterologists, achieving a highly correlated score. This score aimed to provide a standard criterion for quantitative evaluations of CE preparation [68]. Later, Ju et al. created a large-scale semantic segmentation dataset that, combined with a CNN, can differentiate the mucosa’s cleanliness with an accuracy above 94.4% to identify clean mucosa [69].

In January 2023, Ju et al. compared an AI algorithm with the judgment of five gastroenterologists by evaluating 300 video clips with 3000 frames collected from 100 patients. This study reinforces the intra-variability within human judgment and concludes that there was no significant difference between the AI evaluation and human judgment. In addition, AI results were represented on a numerical scale, providing more detailed information [61]. In April 2023, Ribeiro et al. designed a CNN capable of classifying the quality of intestinal preparation in CE. They used a three-level classification scale: excellent, satisfactory, and unsatisfactory, achieving a high accuracy, sensitivity, and specificity of 92.1%, 88.4%, and 93.6%, respectively. The methodology was particularly robust, using images from two different centers, two different SB-CE systems, and a large dataset (CE from 4319 patients, 12,950 images of SB mucosa). This study suggests a high potential for replicating this algorithm in real-time practice [70].

Almost all studies emphasize the importance of validating these scores with different CE systems and bowel preparation types [63,64]. The implementation of CNN algorithms opens the possibility of conducting valid comparative analyses of different preparation regimens. There are already randomized controlled trials that aim to answer this question using computed assessment. Houdeville et al. conducted the first ever research using an ML system to compare two polyethylene glycol (PEG)-based preparations, with and without simethicone, regarding bubble reduction as the primary outcome. Although there was no significant impact on the diagnostic and transit time, there was a marked reduction in the abundance of bubbles over the SB, specifically in the distal ileum. This research was significant as it enhanced the potential role of AI in establishing the gold standard for preparation in SB CE [71].

AI will probably play a role in optimizing cleanliness scores, which is becoming a hot topic in gastroenterology. However, to this day, there has been no incorporation of scores in CE software [10].

### 2.9. Miscellaneous—AI and Hookworms/Functional Bowel disorders

Some studies aim to identify parasites in the GI tract, particularly hookworms. However, the initial studies did not achieve excellent results due to the difficulty faced by the algorithms in differentiating hookworms from other luminal contents [72]. More recently, studies using CNNs achieved better results. Indeed, Gan et al. developed a CNN to detect hookworms in SB CE images automatically. This research reported a sensitivity, specificity, and accuracy of 92.2%, 91.1%, and 91.2%, respectively [73].

There are also studies using AI to evaluate intestinal motor disorders, including the detection and analysis of contractions and the diagnosis of intestinal disorders [74,75].

## 3. Application in Device-Assisted Enteroscopy

CE is guarded as the first-line investigation regarding SB pathology. For instance, device-assisted enteroscopy (DAE) plays a secondary role in the approach of small intestinal lesions, mainly after a positive CE [76].

DAE comprises single- and double-balloon enteroscopy (DBE), plus motorized spiral enteroscopy. DAE enables gastroenterologists to obtain access to the SB in order to obtain sample tissues and perform therapeutic procedures [6,77].

The use of AI to assist in DAE examinations has been scarcely evaluated, and there are only a few articles in the literature. Below is a summary of the main evidence in this regard. Additionally, Table 8 collects the most relevant research regarding AI implementation in SB study using device-assisted enteroscopy in each field of application.

### 3.1. AI and Vascular Lesions

CE and enteroscopy, particularly DAE, have revolutionized the approach to OBGI. The latter possesses a diagnostic nature and accounts for interventional approaches to SB. Although their diagnostic yields for evaluating obscure GI bleeding are similar, the prevailing opinion favors initiating the investigation with CE [76,78]. This preference is attributed to the non-invasive nature of CE, and the diagnostic yield of DBE is significantly enhanced when guided by a previous positive CE study [76,78]. Moreover, observation of the entire SB is desirable to determine the most suitable insertion route for subsequent DAE, as multiple lesions could be identified [79].

As mentioned earlier, the utilization of AI mechanisms for the automatic detection of vascular lesions, in particular angioectasia, has been extensively explored in CE images. Currently, only one study uses an AI-based algorithm to automatically detect angioectasia in DAE images, which provides the initial foundation for future developments.

Saraiva et al., in 2021, developed a DL CNN to differentiate normal mucosa from angioectasia in DAE images. From a pool of 72 patients, 6470 frames were collected, of which 1395 contained angioectasia. Overall, the system had a sensitivity of 89%, a specificity of 97%, and an accuracy of 95%, while achieving reading rates of 155 frames per second. Additionally, it is important to state that the acquired data were retrieved from two different models of DAE, which contributes to its generalization, solving interoperability challenges in real clinical practice [80]. By enhancing the ability to diagnose this type of vascular lesion, AI is expected to improve treatment efficacy, ultimately leading to better patient outcomes and reduced rebleeding rates in the long run.

### 3.2. AI and Ulcers and Erosions

Ulcers and erosions are the most prevalent lesions in the SB, and their etiology is vast, from non-steroid anti-inflammatory drugs to CD or neoplasms. The role of CE in the diagnosis of these conditions is firmly established and is detailed elsewhere in this article. CE’s purely diagnostic nature, coupled with the fact that tissue diagnosis is sometimes required (i.e., excluding infectious enteropathy or the presence of malignant cells), makes DAE a crucial element in the global approach [79].

DAE also plays a part in diagnosing isolated SB CD, a particularly difficult phenotype to diagnose, highly resistant to treatment, and frequently associated with complications that may require therapeutic actions (stricture dilation, hemostasis, etc.) [81]. Indeed, it can clarify the diagnosis with a reported diagnostic yield ranging from 30 to 80% in numerous studies in the literature, despite the limited role in the initial evaluation of these patients [81,82]. Nonetheless, given the high prevalence of stenotic lesions in SB CD, it may be the primary diagnostic tool for patients with CD who are likely to have SB strictures (therefore, a risk of capsule retention) [82,83,84]. Nevertheless, to the best of our knowledge, there is no study regarding the use of AI for diagnosing or monitoring inflammatory burden in SB CD through DAE images.

As far as we know, only one study evaluates AI applicability in automatically detecting ulcers and erosions in DAE. The research study used a total of 6772 DAE images, of which 633 were considered ulcers or erosions. It reported remarkable results regarding sensitivity, specificity, and overall accuracy (88.5%, 99.7%, and 98.7%, respectively), as well as the frame rate capacity. Two different models of DAE were used for data acquisition, once more contributing to its generalization to clinical practice. Another putative advantage of this model is that it allows for a nearly comprehensive panendoscopy AI analysis of the digestive tract [85].

**Table 8:** Summary of studies on AI application in the study of the small bowel using device-assisted enteroscopy, according to the field of application. Ref, reference; Pub, publication year; S, sensitivity; Sp, specificity; Acc, accuracy; AUC, area under the curve; CNN, convolutional neural network.

Author Ref.	Field	Pub. Year	Study Design	Aim	Number of Subjects	Training Dataset	Validation and Testing Dataset	AI Type	Results
Device-Assisted Enteroscopy									
Saraiva et al. [80]	Angioectasia	2021	Retrospective	Automatic detection of angioectasia	72 patients	5392	1348	CNN	S: 88.5%, Sp: 97.1%, Acc: 95.3%, AUC: 0.98
Martins et al. [85]	Ulcers and erosions	2023	Retrospective	Automatic detection of ulcers and erosions	250 patients (6772 images)	6094	678	CNN	S: 89.7%, Sp: 99.5%, Acc: 98.6%
Cardoso et al. [86]	Protruding lesions	2022	Retrospective	Automatic detection of protruding lesions	72 patients	6340	1585	CNN	S: 97.0% Sp: 97.4% Acc: 97.3% AUC 1.00
Mendes et al. [87]	Multiple lesion detection	2024	Retrospective	Automatic detection of multiple clinically relevant lesions	338 exams	36,599	4066	CNN	S: 88.9% Sp: 98.9% Acc: 96.8%

### 3.3. AI and Protuberant Lesions

The role of CE in the diagnosis of SB protuberant lesions has been previously explored. However, because of the inability of CE to provide a tissue diagnosis, perform the resection of lesions, or operate endoscopic therapy, DAE has emerged as a pivotal surrogate, particularly after a positive CE.

Therefore, AI may allow for the detection and further characterization of SB lesions in DAE images when applied to this context. However, this investigation is still in its preliminary stages. Recently, in 2022, Cardoso et al. developed a CNN-based algorithm for the automatic detection of protruding lesions in enteroscopy images (a total of 7925 images). The system achieved a sensitivity, specificity, and accuracy of 97%, 97.4%, and 97.3%, respectively, laying the foundations for future development in this field [86].

### 3.4. AI and Pleomorphic Multi-Lesion Detection 

Device-assisted enteroscopy is the only diagnostic and therapeutic procedure capable of evaluating the entire gastrointestinal tract. Therefore, AI appears as a transformative aid to increment the diagnostic accuracy and cost-effectiveness of this exam in a panendoscopic setting. In January 2024, a Portuguese research group developed a multidevice CNN using 338 DAE exams from two renowned centers. This study achieved a sensitivity of 89%, a specificity of 99%, and an overall accuracy of 96.8% for the diagnosis of multiple clinically relevant lesions in a panendoscopic setting. To the best of our knowledge, this paper is the first to address the detection of pleomorphic multi-lesions, enabling the study not only of the SB but also of other topographies [87].

## 4. Discussion

AI in gastroenterology is already integrated in some fields within clinical practice (namely colonoscopy and endoscopy). However, despite the existing research in CE showing great promise, there is still a long way to go regarding the validation of AI in CE, similarly to what had previously happened with other diagnostic techniques [88].

Prospective studies that restore scientific truth and validate its use as a tool that both enhances diagnostic accuracy and improves time efficiency are required. Nevertheless, some limitations still hinder the execution of such studies in this context. The existence of smaller databases compared to other diagnostic techniques, along with the limited transfer of healthcare data between centers and a lack of rigorous result standardization, makes it challenging to create adequate large sample sizes. Those are essential for planning robust prospective studies that accurately represent the population (reducing selection bias) and avoid model overfitting. Additionally, most studies include images obtained from one single medical center and from a specific capsule brand, highlighting the need to ensure interoperability between systems by creating brand/model-spanning algorithms, which are still rare at the moment. Moreover, until the present day, the majority of studies had only evaluated AI applications at the still-frame level (and not at the video level) and, despite achieving good results, it may not ensure satisfactory performance with full-length videos. It is essential to understand the true practical potential beyond image-level classification. Due to all these factors, the Technology Readiness Level (TRL) of AI algorithms developed in the field of capsule endoscopy is still lower than those available for other techniques. Therefore, before progressing to prospective studies, it is necessary to address the remaining technological challenges and mature the existing technology [88,89].

Another practical concern regards finding a delicate balance between the sensitivity and specificity of developed AI algorithms. Sensitivity is inherently related to diagnostic accuracy, and in turn, specificity is linked to the video-reading time. In other words, it is important for the AI model not only to accurately detect pathological lesions when they are present, but also to correctly identify their absence in frames where no pathological lesions exist. In an extreme scenario of a high false-positive rate (low specificity), the model’s reading time, instead of decreasing, could potentially increase, rendering the algorithm highly time-inefficient. Nevertheless, the current paradigm seems to prioritize initial investment in ensuring higher sensitivity (and so a higher ability to detect lesions), and to concentrate on specificity afterward (ensuring temporal effectiveness).

**Figure 3:**
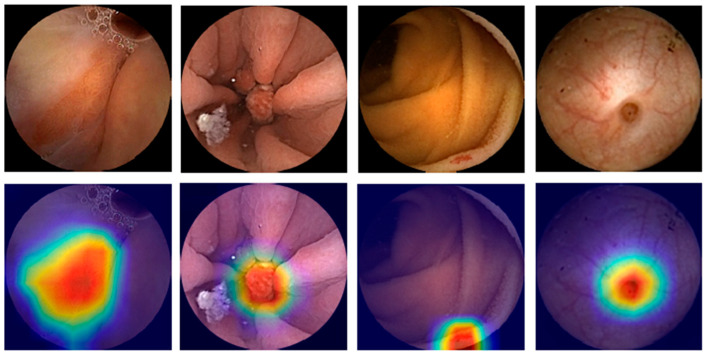
Heatmaps obtained from the application of the convolutional neural network showing pleomorphic lesions identified during small-bowel capsule endoscopy.

**Figure 4:**
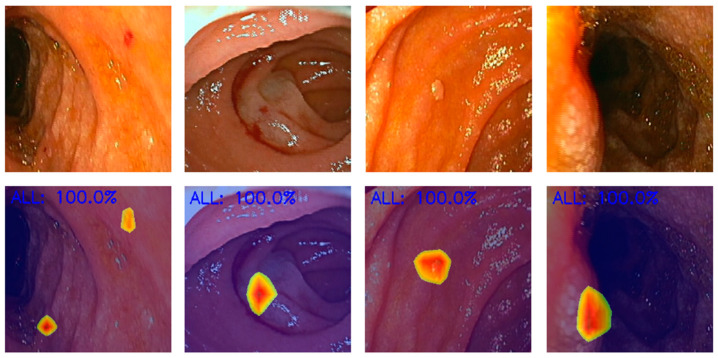
Heatmaps obtained from the application of the convolutional neural network showing pleomorphic lesions identified during device-assisted enteroscopy.

The scarcity of transparency and explainability also remains as a pitfall in AI technology. Indeed, the lack of a clearly understood decision-making mechanism of AI models can reduce humans’ trust in the technology. This problem raises the necessity to develop techniques that allow its users to acquire some understanding from the output provided by AI and to take corrective actions if needed. Attention maps and saliency region identification are good examples of such data explainability capacity techniques (as shown in Figure 3 and Figure 4), but further studies are required to assess their applicability [90,91].

Furthermore, before implementation, it is vital to address the medicolegal problems inherent to the use of AI in healthcare and simultaneously be transparent about the limitations. Indeed, the European Society of Gastrointestinal Endoscopy has issued a position statement regarding the use of artificial intelligence in endoscopy. On the one hand, it emphasizes the potential benefits of integrating AI technologies into endoscopy, but on the other hand, it also highlights the need for careful validation and regulation of AI algorithms, as well as ongoing training for endoscopists to ensure safe and effective implementation [92]. 

From all the evidence previously exposed, AI can correctly assist medical doctors in the clinical decision process, ultimately reducing medical error, even from the most experienced endoscopists, and improving patient outcomes. Additionally, it ensures that procedures will be more accurate and less time-consuming, thus promoting a more sustainable endoscopy. Indeed, reducing the number of procedures performed for diagnostic purposes seems to be the most reliable strategy for an environmentally friendly endoscopic approach, a growing concern in our society [93,94].

AI ensures higher efficiency without necessarily altering the currently available endoscopic techniques, which could result in a smoother adaptation for endoscopists. Hence, AI could alleviate the workload on endoscopic techniques that tends to increase each day and, subsequently, the emotional and physical stress imposed on physicians [5,95].

There is no doubt that AI has the potential to change the current clinical practice by providing more objective scores. Some studies have proven that AI could help to overcome intra-observer variability, reducing the importance of clinical experience. One good example is the potential to help establish a gold-standard score for CE preparation. 

To sum up, the utilization of AI models in gastroenterology still has significant limitations that must be tackled to prevent any harmful impact on patients’ well-being [2,88,96].

## 5. Concluding Remarks

What we expect: The integration of big data and AI in the study of the small bowel could support physicians in expediting the decision-making process, without sacrificing efficiency and ultimately leading to improved healthcare outcomes. 

What we have: The application of AI in our everyday life is already widely notorious. Within the medical field, its performance for studying the SB, both through CE and enteroscopy, attained highly satisfying results, with some articles achieving sensitivity and specificity rates exceeding 90%. 

What is coming: Prospective multicentric research is still missing to corroborate the use of this technology in CE and enteroscopy. Increasing the TRL of AI models available for CE is another future point of focus, and interoperability between a wide range of devices has to be ensured for a proper generalization. The diagnostic process stage at which AI models should be included also has to be defined prior to reading the video (as a screening tool) or during that process. Finally, there is an expectation for a synergy between the two modalities to occur, as enteroscopy can integrate therapeutic interventions to the purely diagnostic nature of CE, further improving the study of the small bowel. The implementation of this technology in both CE and enteroscopy still has a long journey ahead, but the current generation of gastroenterologists will likely witness its inclusion into clinical activity.

## Figures and Tables

**Table 1:** Summary of studies about AI application in the study of OGIB. Ref, reference; Pub, publication year; S, sensitivity; Sp, specificity; Acc, accuracy; AUC, area under the curve; SB, small bowel; GI, gastrointestinal; CE, capsule endoscopy; WCE, wireless capsule endoscopy; SVM, support vector machine; CNN, convolutional neural network; DL, deep learning.

Author Ref.	Field	Pub. Year	Study Design	Aim	Number of Subjects	Training Dataset	Validation and Testing Dataset	AI Type	Results
Small-Bowel Capsule Endoscopy									
Pan et al. [15]	GI bleeding	2010	Retrospective	Detect a bleeding image	150 full videos	-	3172 bleeding frames 11,458 normal frames	CNN	S: 93.1%, Sp: 85.6%
Fu et al. [16]	GI bleeding	2014	Retrospective	Detect a bleeding image	20 different WCE videos	10,000 bleeding and 20,000 non-bleeding frames	10,000 bleeding and 40,000 non-bleeding frames	SVM	S: 99%, Sp: 94%, Acc: 95%
Jia et al. [17]	GI bleeding	2016	Retrospective	Detection of GI bleeding	10,000 images	050 GI bleeding frames and 6150 normal frames	800 GI bleeding frames and 1000 normal frames	CNN	S:99%, Sp:100%
Fan et al. [18]	GI bleeding	2018	Retrospective	Detection of ulcers and erosions in SB mucosa	144 full WCE videos	Ulcers: 2000 images of ulcers and 2400 images of normal mucosa	Ulcers: 500 images of ulcers and 600 images of normal mucosa	CAD DL framework	Ulcers: Acc: 95.2% S: 96.8% Sp 94.8%
						Erosions: 2720 images of erosions and 3200 images of normal mucosa	Erosions: 1500 images of erosions and 4000 images of normal mucosa		Erosions: Acc: 95.3% S: 93.7% Sp 96.0%
Aoki et al. [19]	Obscure GI bleeding	2019	Retrospective	Detection of ulcers and erosions the SB	15,800 images from 180 patients	5360 images of ulcers and erosions (115 patients)	440 images of ulcers and erosions, 10,000 normal images (65 patients)	CNN	AUC of 0.958. Sensitivity of 88.2%, specificity of 90.9%, and accuracy of 90.8%
Wang et al. [20]	Obscure GI bleeding	2019	Retrospective	Detection of ulcers and localization	1504 patients (1076 with ulcers)	15,781 ulcer frames and 17,138 normal frames	4917 ulcer frames and 5007 normal frames	CNN	S: 89.7%, Sp: 90.5%, Acc: 90.1%
Aoki et al. [21]	GI bleeding	2019	Retrospective	Validation of a CNN method as a first reader for ulcer detection	20 full videos	-	-	CNN	Significantly shorter reading time with screening by the CNN, without reducing ng the detection rate of mucosal breaks
Aoki et al. [22]	GI bleeding	2020	Retrospective	Detect GI bleeding	27,847 images from 41 patients	27,847 images (6503 images with blood content from 29 patients and 21,344 normal images from 12 patients)	10,208 images (208 images from 5 patients with blood content and 10,000 images from 20 patients with normal mucosa)	CNN	S: 96.6% Sp: 99.9% Acc: 99.9%
Ghosh et al. [23]	Obscure GI bleeding	2021	-	Detect bleeding zones	-	-	-	CNN	Acc: 94.4%
Afonso et al. [14]	Obscure GI bleeding	2021	Retrospective	Detect blood and hematic residues in the SB lumen	-	Three stages of development. In each stage, the neural architecture was adapted, and the number of CE images increased. In the final stage, 23,190 frames were used.		CNN	S: 98.3% Sp: 98.4%, Acc: 98.2%. Reading time of 186 frames/second)
Saraiva et al. [24]	Obscure GI bleeding	2021	Retrospective	Detection and differentiation of multiple SB lesions with different bleeding potential (Saurin classification)	4319 patients	42,844 images	10,711 images	CNN	S: 88% Sp: 99% Acc: 99%

**Table 4:** Summary of studies on AI application in the study of pleomorphic lesions. Ref, reference; Pub, publication year; S, sensitivity; Acc, accuracy; AUC, area under the curve; SB, small bowel; CE, capsule endoscopy; CNN, convolutional neural network.

Author Ref.	Field	Pub. Year	Study Design	Aim	Number of Subjects	Training Dataset	Validation and Testing Dataset	AI Type	Results
Small-Bowel Capsule Endoscopy									
Ding et al. [43]	Multiple lesion detection	2019	Retrospective	Detect and distingue multiple lesions	6970 patients	158,235 images from 1970 exams	113,268,334 images from 5000 patients	CNN	S near 100%. Mean reading time of 6 min per exam
Otani et al. [44]	Multiple lesion detection	2020	Retrospective	Detect and distingue multiple lesions	167 patients	5526 images (erosions and ulcers, vascular lesions, and tumors) and 34,437 normal images	1247 images.	CNN	AUC: 0.996 for erosions and ulcers, 0.950 for vascular lesions, and 0.950 for tumors
Aoki et al. [45]	Multiple lesion detection	2020	Retrospective	Detect and classify multiple lesions	-	66,028 CE images (44,684 images of lesions and 21,344 normal images)	Full videos from 379 SB CE	CNN	Acc for mucosal breaks, angioectasia, protruding lesions, and blood content were 100%, 97%, 98%, and 100%, respectively
Hwang et al. [42]	Multiple lesion detection	2021	Retrospective	Detect bleeding and ulcerative lesions separately Two models: combined and binary	-	7556 images (half pathological and half normal) from 526 SB CE videos	5760 images (960 abnormal and 4800 normal) from 162 videos	CNN	Both models with high accuracy for lesion detection and localization of the culprit area

**Table 5:** Summary of studies on AI application in the study of celiac disease. Ref, reference; Pub, publication year; S, sensitivity; Sp, specificity; Acc, accuracy; SB, small bowel; CE, capsule endoscopy; VCE, video capsule endoscopy; SVM, support vector machine; CNN, convolutional neural network; ML, machine learning.

Author Ref.	Field	Pub. Year	Study Design	Aim	Number of Subjects	Training Dataset	Validation and Testing Dataset	AI Type	Results
Small-Bowel Capsule Endoscopy									
Ciaccio et al. [8]	Celiac disease	2010	Retrospective	Predict celiac disease based on images of CE SBs	11 patients and 10 controls	six celiac and five control patients’ data	five celiac and five control patients’ data	-	S: 80% Sp: 96% The incremental classifier had 88% S% and 80% Sp%
Zhou et al. [49]	Celiac disease	2017	Retrospective	Evaluate the presence and degree of intestinal villous atrophy	21 patients	six celiac disease patients and five controls	five celiac disease patients and five control patients	CNN	S: 100% Sp: 100% Capable of correlating the Marsh score with CE images
Koh et al. [50]	Celiac disease	2018	Retrospective	Identify patients with celiac disease	13 control subjects and 13 celiac patients	-	-	SVM	S: 88.4% Sp: 84.6% Acc: 86.5%
Wang et al. [51]	Celiac disease	2020		Identify patients with celiac disease	107 exams	1100 images with healthy mucosa and 1040 lesion images		CNN	S: 97.2% Sp: 95.6% Acc: 95.9%
Stoleru et al. [52]	Celiac disease	2022	Retrospective	Diagnose celiac disease with CE images, without complex algorithm	105 exams	51 videos (of 100 frames)	51 videos (of 100 frames)	ML	Acc: 94.1%
Zammit et al. [53]	Celiac disease	2023	Retrospective	Evaluate and grade celiac disease severity, compare with expert classification	-	334,080 frames from 35 patients with celiac disease. 110,579 frames from 13 patients without celiac disease	63 VCE videos from 63 patients with celiac disease	ML	Strong correlation between celiac severity scores provided by the algorithm and the average expert reader scores

**Table 6:** Summary of studies on AI application in the study of inflammatory bowel activity. Ref, reference; Pub, publication year; Acc, accuracy; AUC, area under the curve; SB, small bowel; CE, capsule endoscopy; CNN, convolutional neural network; DL, deep learning.

Author Ref.	Field	Pub. Year	Study Design	Aim	Number of Subjects	Training Dataset	Validation and Testing dataset	AI Type	Results
Small-Bowel Capsule Endoscopy									
Klang et al. [56]	Inflammatory bowel disease	2019	Retrospective	Detection of SB ulcers in Crohn’s disease patients	17,640 CE images from 49 patients	-	-	CNN	Acc over 95% AUC over 0.94
Barash et al. [58]	Inflammatory bowel disease	2020	Retrospective	Detect and grade the severity of ulcers in Crohn’s disease and access the inter-reader variability and the agreement of two experts and the AI method	17,640 CE images from 49 patients	Pre-train with 17,640 CE images (7391 images with mucosal ulcers and 10,249 images of normal mucosa). Train with 1242 images.	248 images	CNN	Overall agreement between the consensus reading and the automatic algorithm of 67% but an inter-reader agreement of only 31%
Klang et al. [59]	Inflammatory bowel disease	2020	Retrospective	Detect strictures in CE images of Crohn’s disease patients	27,892 CE images	-	-	DL	Acc: 93.5% Excellent discrimination between strictures, normal mucosa, and different grades of ulcers

**Table 7:** Summary of studies on AI application in the study of small-bowel cleansing. Ref, reference; Pub, publication year; S, sensitivity; Sp, specificity; Acc, accuracy; SB, small bowel; CE, capsule endoscopy; VCE, video capsule endoscopy; CNN, convolutional neural network; DL, deep learning; ML, machine learning; CAC, computer assessment of cleansing.

Author Ref.	Field	Pub. Year	Study Design	Aim	Number of Subjects	Training Dataset	Validation and Testing Dataset	AI Type	Results
Small-Bowel Capsule Endoscopy									
Van Weyen-berg et al. [63]	SB cleanliness	2011	Retrospective	Design an objective score of quality of SB visualization—computer assessment of cleansing (CAC) score	40 VCE segments from 10 VCE studies	-	-	Computer evaluation	Show feasibility of using the CAC score in the assessment of the quality of intestinal preparation in PillCam^®^ CE system
Ponte et al. [64]	SB cleanliness	2016	Retrospective	Adapt the CAC score to the Mirocam^®^ CE system	30 VCE	-	-	Computer evaluation	Results slightly inferior to those of Van Weyenberg but significant
Abou Ali et al. [65]	SB cleanliness	2018	Retrospective	Develop and validate a CAC score at the image level by defining the threshold for an adequate SB visualization	33 VCE	-	-	Computer evaluation	S: 91.3% Sp: 94.7%
Oumrani et al. [66]	SB cleanliness	2019	Retrospective	Access the adequacy of SB mucosa visualization	600 frames	500 frames	100 frames	ML	S: 90.0% Sp: 87.7%
Noorda et al. [60]	SB cleanliness	2020	Retrospective	Access the adequacy of SB mucosa visualization with an intuitive scale	Images from 35 VCE	26,746 clean patches and 28,547 dirty patches	854 frames extracted from 30 different CE videos	CNN	Acc: 95.2%
Leenhardt et al. [67]	SB cleanliness	2020	Retrospective	Access SB mucosa visualization	186 VCE	600 still frames	two independent 78-video subsets	CNN	S: 90.3% Sp: 83.3% Acc: 89.7%
Nam et al. [68]	SB cleanliness	2021	Retrospective	Provide an objective score for quantitative evaluation of CE cleanliness	168 CE exams	2500 frames	1000 frames	DL	Score had high correlation with assessment by CE experts
Ju et al. [61]	SB cleanliness	2023	Retrospective	Compare the detection of clean mucosal areas in CE using human judgment versus AI	13,233 images from 512 CE exams	2319 images from 12 patients	10,914 images from 500 patients	CNN	Intra-variability within human judgment. AI judgment was consistent with the five gastroenterologists’ judgements
Ju et al. [69]	SB cleanliness	2022	Retrospective	Create a large-scale semantic segmentation dataset and combine with a CNN to evaluate SB cleanliness	10,033 images from 179 CE studies	7988 images from 169 patients	2045 images from 10 patients	CNN	Acc above 94%
Ribeiro et al. [70]	SB cleanliness	2023	Retrospective	Assess the quality of intestinal preparation in CE	4319 patients	12,159 images	791 images	CNN	S: 88.4% Sp: 93.6% Acc: 92.1%

## Data Availability

Not applicable.

## References

[1] Yang Y.C., Islam S.U., Noor A., Khan S., Afsar W., Nazir S. (2021). Influential Usage of Big Data and Artificial Intelligence in Healthcare. Comput. Math. Methods Med..

[2] Mascarenhas M., Afonso J., Andrade P., Cardoso H., Macedo G. (2021). Artificial intelligence and capsule endoscopy: Unravelling the future. Ann. Gastroenterol..

[3] Catlow J., Bray B., Morris E., Rutter M. (2022). Power of big data to improve patient care in gastroenterology. Frontline Gastroenterol..

[4] Pannala R., Krishnan K., Melson J., Parsi M.A., Schulman A.R., Sullivan S., Trikudanathan G., Trindade A.J., Watson R.R., Maple J.T. (2020). Artificial intelligence in gastrointestinal endoscopy. VideoGIE.

[5] Okagawa Y., Abe S., Yamada M., Oda I., Saito Y. (2022). Artificial Intelligence in Endoscopy. Dig. Dis. Sci..

[6] Lee H.H., Kim J.S., Goong H.J., Lee S.H., Oh E.H., Park J., Kim M.C., Nam K., Yang Y.J., Kim T.J. (2023). Use of device-assisted enteroscopy in small bowel disease: An expert consensus statement by the Korean Association for the Study of Intestinal Diseases. Intest. Res..

[7] Cortegoso Valdivia P., Skonieczna-Zydecka K., Elosua A., Sciberras M., Piccirelli S., Rullan M., Tabone T., Gawel K., Stachowski A., Leminski A. (2022). Indications, Detection, Completion and Retention Rates of Capsule Endoscopy in Two Decades of Use: A Systematic Review and Meta-Analysis. Diagnostics.

[8] Ciaccio E.J., Tennyson C.A., Bhagat G., Lewis S.K., Green P.H. (2010). Classification of videocapsule endoscopy image patterns: Comparative analysis between patients with celiac disease and normal individuals. Biomed. Eng. Online.

[9] Majtner T., Brodersen J.B., Herp J., Kjeldsen J., Halling M.L., Jensen M.D. (2021). A deep learning framework for autonomous detection and classification of Crohn’s disease lesions in the small bowel and colon with capsule endoscopy. Endosc. Int. Open.

[10] Mascarenhas M., Cardoso H., Macedo G. (2023). Artificial Intelligence in Capsule Endoscopy: A Gamechanger for a Groundbreaking Technique.

[11] Awadie H., Zoabi A., Gralnek I.M. (2022). Obscure-overt gastrointestinal bleeding: A review. Pol. Arch. Intern. Med..

[12] Patel A., Vedantam D., Poman D.S., Motwani L., Asif N. (2022). Obscure Gastrointestinal Bleeding and Capsule Endoscopy: A Win-Win Situation or Not?. Cureus.

[13] Jackson C.S., Strong R. (2017). Gastrointestinal Angiodysplasia: Diagnosis and Management. Gastrointest. Endosc. Clin. N. Am..

[14] Afonso J., Saraiva M.M., Ferreira J.P.S., Ribeiro T., Cardoso H., Macedo G. (2021). Performance of a convolutional neural network for automatic detection of blood and hematic residues in small bowel lumen. Dig. Liver Dis..

[15] Pan G., Yan G., Qiu X., Cui J. (2011). Bleeding detection in Wireless Capsule Endoscopy based on Probabilistic Neural Network. J. Med. Syst..

[16] Fu Y., Zhang W., Mandal M., Meng M.Q. (2014). Computer-aided bleeding detection in WCE video. IEEE J. Biomed. Health Inf..

[17] Xiao J., Meng M.Q. (2016). A deep convolutional neural network for bleeding detection in Wireless Capsule Endoscopy images. Annu. Int. Conf. IEEE Eng. Med. Biol. Soc..

[18] Fan S., Xu L., Fan Y., Wei K., Li L. (2018). Computer-aided detection of small intestinal ulcer and erosion in wireless capsule endoscopy images. Phys. Med. Biol..

[19] Aoki T., Yamada A., Aoyama K., Saito H., Tsuboi A., Nakada A., Niikura R., Fujishiro M., Oka S., Ishihara S. (2019). Automatic detection of erosions and ulcerations in wireless capsule endoscopy images based on a deep convolutional neural network. Gastrointest. Endosc..

[20] Wang S., Xing Y., Zhang L., Gao H., Zhang H. (2019). A systematic evaluation and optimization of automatic detection of ulcers in wireless capsule endoscopy on a large dataset using deep convolutional neural networks. Phys. Med. Biol..

[21] Aoki T., Yamada A., Aoyama K., Saito H., Fujisawa G., Odawara N., Kondo R., Tsuboi A., Ishibashi R., Nakada A. (2020). Clinical usefulness of a deep learning-based system as the first screening on small-bowel capsule endoscopy reading. Dig. Endosc..

[22] Aoki T., Yamada A., Kato Y., Saito H., Tsuboi A., Nakada A., Niikura R., Fujishiro M., Oka S., Ishihara S. (2020). Automatic detection of blood content in capsule endoscopy images based on a deep convolutional neural network. J. Gastroenterol. Hepatol..

[23] Ghosh T., Chakareski J. (2021). Deep Transfer Learning for Automated Intestinal Bleeding Detection in Capsule Endoscopy Imaging. J. Digit. Imaging.

[24] Mascarenhas Saraiva M.J., Afonso J., Ribeiro T., Ferreira J., Cardoso H., Andrade A.P., Parente M., Natal R., Mascarenhas Saraiva M., Macedo G. (2021). Deep learning and capsule endoscopy: Automatic identification and differentiation of small bowel lesions with distinct haemorrhagic potential using a convolutional neural network. BMJ Open Gastroenterol..

[25] Vieira P.M., Silva C.P., Costa D., Vaz I.F., Rolanda C., Lima C.S. (2019). Automatic Segmentation and Detection of Small Bowel Angioectasias in WCE Images. Ann. Biomed. Eng..

[26] Vieira P.M., Goncalves B., Goncalves C.R., Lima C.S. (2016). Segmentation of angiodysplasia lesions in WCE images using a MAP approach with Markov Random Fields. Annu. Int. Conf. IEEE Eng. Med. Biol. Soc..

[27] Noya F., Alvarez-Gonzalez M.A., Benitez R. (2017). Automated angiodysplasia detection from wireless capsule endoscopy. Annu. Int. Conf. IEEE Eng. Med. Biol. Soc..

[28] Leenhardt R., Vasseur P., Li C., Saurin J.C., Rahmi G., Cholet F., Becq A., Marteau P., Histace A., Dray X. (2019). A neural network algorithm for detection of GI angiectasia during small-bowel capsule endoscopy. Gastrointest. Endosc..

[29] Tsuboi A., Oka S., Aoyama K., Saito H., Aoki T., Yamada A., Matsuda T., Fujishiro M., Ishihara S., Nakahori M. (2020). Artificial intelligence using a convolutional neural network for automatic detection of small-bowel angioectasia in capsule endoscopy images. Dig. Endosc..

[30] Chu Y., Huang F., Gao M., Zou D.W., Zhong J., Wu W., Wang Q., Shen X.N., Gong T.T., Li Y.Y. (2023). Convolutional neural network-based segmentation network applied to image recognition of angiodysplasias lesion under capsule endoscopy. World J. Gastroenterol..

[31] Van de Bruaene C., De Looze D., Hindryckx P. (2015). Small bowel capsule endoscopy: Where are we after almost 15 years of use?. World J. Gastrointest. Endosc..

[32] Mascarenhas Saraiva M., Afonso J., Ribeiro T., Ferreira J., Cardoso H., Andrade P., Gonçalves R., Cardoso P., Parente M., Jorge R. (2023). Artificial intelligence and capsule endoscopy: Automatic detection of enteric protruding lesions using a convolutional neural network. Rev. Esp. Enferm. Dig..

[33] Barbosa D.J., Ramos J., Lima C.S. (2008). Detection of small bowel tumors in capsule endoscopy frames using texture analysis based on the discrete wavelet transform. Annu. Int. Conf. IEEE Eng. Med. Biol. Soc..

[34] Barbosa D.C., Roupar D.B., Ramos J.C., Tavares A.C., Lima C.S. (2012). Automatic small bowel tumor diagnosis by using multi-scale wavelet-based analysis in wireless capsule endoscopy images. Biomed. Eng. Online.

[35] Li B., Meng M.Q., Xu L. (2009). A comparative study of shape features for polyp detection in wireless capsule endoscopy images. Annu. Int. Conf. IEEE Eng. Med. Biol. Soc..

[36] Li B.P., Meng M.Q. (2012). Comparison of several texture features for tumor detection in CE images. J. Med. Syst..

[37] Li B., Meng M.Q. (2012). Tumor recognition in wireless capsule endoscopy images using textural features and SVM-based feature selection. IEEE Trans. Inf. Technol. Biomed..

[38] Vieira P.M., Freitas N.R., Valente J., Vaz I.F., Rolanda C., Lima C.S. (2020). Automatic detection of small bowel tumors in wireless capsule endoscopy images using ensemble learning. Med. Phys..

[39] Vieira P.M., Ramos J., Lima C.S. (2015). Automatic detection of small bowel tumors in endoscopic capsule images by ROI selection based on discarded lightness information. Annu. Int. Conf. IEEE Eng. Med. Biol. Soc..

[40] Yuan Y., Meng M.Q. (2017). Deep learning for polyp recognition in wireless capsule endoscopy images. Med. Phys..

[41] Saito H., Aoki T., Aoyama K., Kato Y., Tsuboi A., Yamada A., Fujishiro M., Oka S., Ishihara S., Matsuda T. (2020). Automatic detection and classification of protruding lesions in wireless capsule endoscopy images based on a deep convolutional neural network. Gastrointest. Endosc..

[42] Hwang Y., Lee H.H., Park C., Tama B.A., Kim J.S., Cheung D.Y., Chung W.C., Cho Y.S., Lee K.M., Choi M.G. (2021). Improved classification and localization approach to small bowel capsule endoscopy using convolutional neural network. Dig. Endosc..

[43] Ding Z., Shi H., Zhang H., Meng L., Fan M., Han C., Zhang K., Ming F., Xie X., Liu H. (2019). Gastroenterologist-Level Identification of Small-Bowel Diseases and Normal Variants by Capsule Endoscopy Using a Deep-Learning Model. Gastroenterology.

[44] Otani K., Nakada A., Kurose Y., Niikura R., Yamada A., Aoki T., Nakanishi H., Doyama H., Hasatani K., Sumiyoshi T. (2020). Automatic detection of different types of small-bowel lesions on capsule endoscopy images using a newly developed deep convolutional neural network. Endoscopy.

[45] Aoki T., Yamada A., Kato Y., Saito H., Tsuboi A., Nakada A., Niikura R., Fujishiro M., Oka S., Ishihara S. (2021). Automatic detection of various abnormalities in capsule endoscopy videos by a deep learning-based system: A multicenter study. Gastrointest. Endosc..

[46] Vieira P.M., Freitas N.R., Lima V.B., Costa D., Rolanda C., Lima C.S. (2021). Multi-pathology detection and lesion localization in WCE videos by using the instance segmentation approach. Artif. Intell. Med..

[47] Wang C., Luo Z., Liu X., Bai J., Liao G. (2018). Organic Boundary Location Based on Color-Texture of Visual Perception in Wireless Capsule Endoscopy Video. J. Healthc. Eng..

[48] Raiteri A., Granito A., Giamperoli A., Catenaro T., Negrini G., Tovoli F. (2022). Current guidelines for the management of celiac disease: A systematic review with comparative analysis. World J. Gastroenterol..

[49] Zhou T., Han G., Li B.N., Lin Z., Ciaccio E.J., Green P.H., Qin J. (2017). Quantitative analysis of patients with celiac disease by video capsule endoscopy: A deep learning method. Comput. Biol. Med..

[50] Koh J.E.W., Hagiwara Y., Oh S.L., Tan J.H., Ciaccio E.J., Green P.H., Lewis S.K., Acharya U.R. (2018). Automated diagnosis of celiac disease using DWT and nonlinear features with video capsule endoscopy images. Future Gener. Comput. Syst..

[51] Wang X., Qian H., Ciaccio E.J., Lewis S.K., Bhagat G., Green P.H., Xu S., Huang L., Gao R., Liu Y. (2020). Celiac disease diagnosis from videocapsule endoscopy images with residual learning and deep feature extraction. Comput. Methods Programs Biomed..

[52] Stoleru C.A., Dulf E.H., Ciobanu L. (2022). Automated detection of celiac disease using Machine Learning Algorithms. Sci. Rep..

[53] Chetcuti Zammit S., McAlindon M.E., Greenblatt E., Maker M., Siegelman J., Leffler D.A., Yardibi O., Raunig D., Brown T., Sidhu R. (2023). Quantification of Celiac Disease Severity Using Video Capsule Endoscopy: A Comparison of Human Experts and Machine Learning Algorithms. Curr. Med. Imaging.

[54] Goran L., Negreanu A.M., Stemate A., Negreanu L. (2018). Capsule endoscopy: Current status and role in Crohn’s disease. World J. Gastrointest. Endosc..

[55] Lamb C.A., Kennedy N.A., Raine T., Hendy P.A., Smith P.J., Limdi J.K., Hayee B., Lomer M.C.E., Parkes G.C., Selinger C. (2019). British Society of Gastroenterology consensus guidelines on the management of inflammatory bowel disease in adults. Gut.

[56] Klang E., Barash Y., Margalit R.Y., Soffer S., Shimon O., Albshesh A., Ben-Horin S., Amitai M.M., Eliakim R., Kopylov U. (2020). Deep learning algorithms for automated detection of Crohn’s disease ulcers by video capsule endoscopy. Gastrointest. Endosc..

[57] Takenaka K., Kawamoto A., Okamoto R., Watanabe M., Ohtsuka K. (2022). Artificial intelligence for endoscopy in inflammatory bowel disease. Intest. Res..

[58] Barash Y., Azaria L., Soffer S., Margalit Yehuda R., Shlomi O., Ben-Horin S., Eliakim R., Klang E., Kopylov U. (2021). Ulcer severity grading in video capsule images of patients with Crohn’s disease: An ordinal neural network solution. Gastrointest. Endosc..

[59] Klang E., Grinman A., Soffer S., Margalit Yehuda R., Barzilay O., Amitai M.M., Konen E., Ben-Horin S., Eliakim R., Barash Y. (2021). Automated Detection of Crohn’s Disease Intestinal Strictures on Capsule Endoscopy Images Using Deep Neural Networks. J. Crohns Colitis.

[60] Noorda R., Nevárez A., Colomer A., Pons Beltrán V., Naranjo V. (2020). Automatic evaluation of degree of cleanliness in capsule endoscopy based on a novel CNN architecture. Sci. Rep..

[61] Ju J., Oh H.S., Lee Y.J., Jung H., Lee J.H., Kang B., Choi S., Kim J.H., Kim K.O., Chung Y.J. (2023). Clean mucosal area detection of gastroenterologists versus artificial intelligence in small bowel capsule endoscopy. Medicine.

[62] Rosa B., Margalit-Yehuda R., Gatt K., Sciberras M., Girelli C., Saurin J.C., Valdivia P.C., Cotter J., Eliakim R., Caprioli F. (2021). Scoring systems in clinical small-bowel capsule endoscopy: All you need to know!. Endosc. Int. Open.

[63] Van Weyenberg S.J., De Leest H.T., Mulder C.J. (2011). Description of a novel grading system to assess the quality of bowel preparation in video capsule endoscopy. Endoscopy.

[64] Ponte A., Pinho R., Rodrigues A., Silva J., Rodrigues J., Carvalho J. (2016). Validation of the computed assessment of cleansing score with the Mirocam^®^ system. Rev. Esp. Enferm. Dig..

[65] Abou Ali E., Histace A., Camus M., Gerometta R., Becq A., Pietri O., Nion-Larmurier I., Li C., Chaput U., Marteau P. (2018). Development and validation of a computed assessment of cleansing score for evaluation of quality of small-bowel visualization in capsule endoscopy. Endosc. Int. Open.

[66] Oumrani S., Histace A., Abou Ali E., Pietri O., Becq A., Houist G., Nion-Larmurier I., Camus M., Florent C., Dray X. (2019). Multi-criterion, automated, high-performance, rapid tool for assessing mucosal visualization quality of still images in small bowel capsule endoscopy. Endosc. Int. Open.

[67] Leenhardt R., Souchaud M., Houist G., Le Mouel J.P., Saurin J.C., Cholet F., Rahmi G., Leandri C., Histace A., Dray X. (2021). A neural network-based algorithm for assessing the cleanliness of small bowel during capsule endoscopy. Endoscopy.

[68] Nam J.H., Hwang Y., Oh D.J., Park J., Kim K.B., Jung M.K., Lim Y.J. (2021). Development of a deep learning-based software for calculating cleansing score in small bowel capsule endoscopy. Sci. Rep..

[69] Ju J.W., Jung H., Lee Y.J., Mun S.W., Lee J.H. (2022). Semantic Segmentation Dataset for AI-Based Quantification of Clean Mucosa in Capsule Endoscopy. Medicina.

[70] Ribeiro T., Mascarenhas Saraiva M.J., Afonso J., Cardoso P., Mendes F., Martins M., Andrade A.P., Cardoso H., Mascarenhas Saraiva M., Ferreira J. (2023). Design of a Convolutional Neural Network as a Deep Learning Tool for the Automatic Classification of Small-Bowel Cleansing in Capsule Endoscopy. Medicina.

[71] Houdeville C., Leenhardt R., Souchaud M., Velut G., Carbonell N., Nion-Larmurier I., Nuzzo A., Histace A., Marteau P., Dray X. (2022). Evaluation by a Machine Learning System of Two Preparations for Small Bowel Capsule Endoscopy: The BUBS (Burst Unpleasant Bubbles with Simethicone) Study. J. Clin. Med..

[72] Wu X., Chen H., Gan T., Chen J., Ngo C.W., Peng Q. (2016). Automatic Hookworm Detection in Wireless Capsule Endoscopy Images. IEEE Trans. Med. Imaging.

[73] Gan T., Yang Y., Liu S., Zeng B., Yang J., Deng K., Wu J., Yang L. (2021). Automatic Detection of Small Intestinal Hookworms in Capsule Endoscopy Images Based on a Convolutional Neural Network. Gastroenterol. Res. Pr..

[74] Spyridonos P., Vilariño F., Vitrià J., Azpiroz F., Radeva P. (2006). Anisotropic feature extraction from endoluminal images for detection of intestinal contractions. Med. Image Comput. Comput. Assist. Interv..

[75] Malagelada C., De Iorio F., Azpiroz F., Accarino A., Segui S., Radeva P., Malagelada J.R. (2008). New insight into intestinal motor function via noninvasive endoluminal image analysis. Gastroenterology.

[76] Teshima C.W., Kuipers E.J., van Zanten S.V., Mensink P.B. (2011). Double balloon enteroscopy and capsule endoscopy for obscure gastrointestinal bleeding: An updated meta-analysis. J. Gastroenterol. Hepatol..

[77] Pennazio M., Rondonotti E., Despott E.J., Dray X., Keuchel M., Moreels T., Sanders D.S., Spada C., Carretero C., Cortegoso Valdivia P. (2023). Small-bowel capsule endoscopy and device-assisted enteroscopy for diagnosis and treatment of small-bowel disorders: European Society of Gastrointestinal Endoscopy (ESGE) Guideline-Update 2022. Endoscopy.

[78] Sun B., Rajan E., Cheng S., Shen R., Zhang C., Zhang S., Wu Y., Zhong J. (2006). Diagnostic yield and therapeutic impact of double-balloon enteroscopy in a large cohort of patients with obscure gastrointestinal bleeding. Am. J. Gastroenterol..

[79] Sakai E., Ohata K., Nakajima A., Matsuhashi N. (2019). Diagnosis and therapeutic strategies for small bowel vascular lesions. World J. Gastroenterol..

[80] Mascarenhas Saraiva M., Ribeiro T., Afonso J., Andrade P., Cardoso P., Ferreira J., Cardoso H., Macedo G. (2021). Deep Learning and Device-Assisted Enteroscopy: Automatic Detection of Gastrointestinal Angioectasia. Medicina.

[81] Yen H.H., Chang C.W., Chou J.W., Wei S.C. (2017). Balloon-Assisted Enteroscopy and Capsule Endoscopy in Suspected Small Bowel Crohn’s Disease. Clin. Endosc..

[82] Jang H.J., Choi M.H., Eun C.S., Choi H., Choi K.Y., Park D.I., Park J.H., Chang D.K., Kim J.O., Ko B.M. (2014). Clinical usefulness of double balloon enteroscopy in suspected Crohn’s disease: The KASID multi-center trial. Hepatogastroenterology.

[83] Rahman A., Ross A., Leighton J.A., Schembre D., Gerson L., Lo S.K., Waxman I., Dye C., Semrad C. (2015). Double-balloon enteroscopy in Crohn’s disease: Findings and impact on management in a multicenter retrospective study. Gastrointest. Endosc..

[84] Bourreille A., Ignjatovic A., Aabakken L., Loftus E.V., Eliakim R., Pennazio M., Bouhnik Y., Seidman E., Keuchel M., Albert J.G. (2009). Role of small-bowel endoscopy in the management of patients with inflammatory bowel disease: An international OMED-ECCO consensus. Endoscopy.

[85] Martins M., Mascarenhas M., Afonso J., Ribeiro T., Cardoso P., Mendes F., Cardoso H., Andrade P., Ferreira J., Macedo G. (2023). Deep-Learning and Device-Assisted Enteroscopy: Automatic Panendoscopic Detection of Ulcers and Erosions. Medicina.

[86] Cardoso P., Saraiva M.M., Afonso J., Ribeiro T., Andrade P., Ferreira J., Cardoso H., Macedo G. (2022). Artificial Intelligence and Device-Assisted Enteroscopy: Automatic Detection of Enteric Protruding Lesions Using a Convolutional Neural Network. Clin. Transl. Gastroenterol..

[87] Mendes F., Mascarenhas M., Ribeiro T., Afonso J., Cardoso P., Martins M., Cardoso H., Andrade P., Ferreira J.P.S., Mascarenhas Saraiva M. (2024). Artificial Intelligence and Panendoscopy—Automatic Detection of Clinically Relevant Lesions in Multibrand Device-Assisted Enteroscopy. Cancers.

[88] Leenhardt R., Koulaouzidis A., Histace A., Baatrup G., Beg S., Bourreille A., de Lange T., Eliakim R., Iakovidis D., Dam Jensen M. (2022). Key research questions for implementation of artificial intelligence in capsule endoscopy. Ther. Adv. Gastroenterol..

[89] Lavin A., Gilligan-Lee C.M., Visnjic A., Ganju S., Newman D., Ganguly S., Lange D., Baydin A.G., Sharma A., Gibson A. (2022). Technology readiness levels for machine learning systems. Nat. Commun..

[90] Meher D., Gogoi M., Bharali P., Anirvan P., Singh S.P. (2020). Artificial intelligence in small bowel endoscopy: Current perspectives and future directions. J. Dig. Endosc..

[91] Leenhardt R., Fernandez-Urien Sainz I., Rondonotti E., Toth E., Van de Bruaene C., Baltes P., Rosa B.J., Triantafyllou K., Histace A., Koulaouzidis A. (2021). PEACE: Perception and Expectations toward Artificial Intelligence in Capsule Endoscopy. J. Clin. Med..

[92] Messmann H., Bisschops R., Antonelli G., Libânio D., Sinonquel P., Abdelrahim M., Ahmad O.F., Areia M., Bergman J., Bhandari P. (2022). Expected value of artificial intelligence in gastrointestinal endoscopy: European Society of Gastrointestinal Endoscopy (ESGE) Position Statement. Endoscopy.

[93] Lee J., Wallace M.B. (2021). State of the Art: The Impact of Artificial Intelligence in Endoscopy 2020. Curr. Gastroenterol. Rep..

[94] Mascarenhas M., Ribeiro T., Afonso J., Mendes F., Cardoso P., Martins M., Ferreira J., Macedo G. (2023). Smart Endoscopy Is Greener Endoscopy: Leveraging Artificial Intelligence and Blockchain Technologies to Drive Sustainability in Digestive Health Care. Diagnostics.

[95] Namikawa K., Hirasawa T., Yoshio T., Fujisaki J., Ozawa T., Ishihara S., Aoki T., Yamada A., Koike K., Suzuki H. (2020). Utilizing artificial intelligence in endoscopy: A clinician’s guide. Expert. Rev. Gastroenterol. Hepatol..

[96] Mascarenhas M., Afonso J., Ribeiro T., Andrade P., Cardoso H., Macedo G. (2023). The Promise of Artificial Intelligence in Digestive Healthcare and the Bioethics Challenges It Presents. Medicina.

